# *In Silico* Pharmacogenetics CYP2D6 Study Focused on the Pharmacovigilance of Herbal Antidepressants

**DOI:** 10.3389/fphar.2020.00683

**Published:** 2020-05-13

**Authors:** Charleen G. Don, Martin Smieško

**Affiliations:** Computational Pharmacy Group, Department of Pharmaceutical Sciences, University of Basel, Basel, Switzerland

**Keywords:** CYP2D6 inhibition, natural products, molecular modeling, pharmacogenetics, adverse reactions, antidepressants, drug safety

## Abstract

The annual increase in depression worldwide together with an upward trend in the use of alternative medicine as treatment asks for developing reliable safety profiles of herbal based medicine. A considerable risk on adverse reactions exists when herbal remedies are combined with prescription medication. Around 25% of the drugs, including many antidepressants, depend on the activity of CYP2D6 for their metabolism and corresponding efficacy. Therefore, probing CYP2D6 inhibition by the active substances in herbal based medicine within the wild-type enzyme and clinically relevant allelic variants is crucial to avoid toxicity issues. In this *in silico* study several compounds with herbal origin suggested to have antidepressant activity were analyzed on their CYP2D6 wild-type and CYP2D6*53 inhibition potential using molecular docking. In addition, several pharmacokinetic properties were evaluated to assess their probability to cross the blood brain barrier and subsequently reach sufficient brain bioavailability for the modulation of central nervous system targets as well as characteristics which may hint toward potential safety issues.

## Introduction

Depression as a mood disorder has significantly increased (~18%) during the last decade affecting over 300 million people as estimated by the World Health Organization (WHO) (Bashar et al., 2019). Concurrently, an upward trend is seen in the use of antidepressants worldwide (Abbing-Karahagopian et al., 2014; Forns et al., 2019; Iacobucci, 2019) in parallel with the use of complementary and alternative medicine (CAM) (Kemper et al., 2013; Asher et al., 2017; Kemppainen et al., 2018). A recent study reported that in Europe (21 surveyed countries) 30% of the affected individuals used CAM to treat depression of which 10% included herbal medicine (natural products) (Kemppainen et al., 2018). Antidepressants as neurotherapeutics must be able to cross the blood-brain barrier (BBB) in order to modulate the function of the central nervous system (CNS). The compound exchange between the blood and the nervous tissue is strictly controlled by a continuous layer of endothelial cells which are kept together by tight junctions with a total estimated surface of around 20 m^2^ (Wong et al., 2013). From a medicinal chemistry perspective, physiochemical properties of CNS modulators (e.g. molecular weight, lipophilicity, hydrogen acceptor, and donor counts) therefore need to fall within a narrower range compared to regular drugs in order to be absorbed, dissolved and permeate across the brain-barrier to reach the CNS (passive transport, non-energy required). More hydrophilic modulators (or other compounds) may still cross the barrier through carrier systems, transporters, or endocytosis (active-transport, ATP required) (Sanchez-Covarrubias et al., 2014; Vendel et al., 2019). However, good BBB permeation does not guarantee that the efficacious concentration of the compound in the CNS compartment is reached (Dong, 2018). For the design of a novel neurotherapeutically acting drug or natural-derived compound, predicting its BBB permeability, partitioning, P450 cytochrome association (inhibitor/substrate/none) and off-target binding in the early development stage is essential in order to develop a compound that will have sufficient brain bioavailability ensuring that the desirable therapeutic effect is acquired and maintained at a concentration sufficient to modulate the CNS system without inducing adverse reactions.

Safety profiles of antidepressant drugs are studied by many groups and unfortunately are known to include also serious side effects (Santarsieri and Schwartz, 2015; Carvalho et al., 2016; Wang et al., 2018). The safety profiles of herbal medicine products (from plant sources) are much less studied despite of being frequently concomitantly administered with another (synthetic) drug and bear a considerable risk for herbal-induced toxicity issues (Ekor, 2014; Vahabi and Eatemadi, 2016; Singh and Zhao, 2017). Herbal-drug interactions (HDI) can lead to harmful adverse events as they may alter the pharmacokinetics (PK; adsorption, distribution, metabolism, or excretion) and pharmacodynamics (PD; pharmacological effect is changed in a synergetic, additive, or antagonistic way) profile of the concomitantly administered drug (Hermann and Richter, 2012).

The prediction of interactions of the natural compounds with one major class of drug metabolizing enzymes, cytochromes P450, especially the family member CYP2D6, is of particular interest. This isoform is responsible for biotransformation of about 25% of all the marketed drugs, and displays a very high polymorphism rate (Zhou, 2009; Don and Smieško, 2018). Inhibition or induction of CYP2D6 metabolism can alter the pharmacokinetic profile of the concomitantly administered drug and potentially can lead to toxicity or affect the drug efficacy (Singh and Zhao, 2017). During the catalytic reaction, interference of the substrate binding to the heme, the binding of molecular oxygen, or the biotransformation step in which the substrate is oxidized are prone to induce adverse reactions upon inhibition (Correia Ortiz de Montellano, 2015). Based on these three interference mechanisms, inhibitors can be divided into three categories; i) reversible binders (competitive or noncompetitive), ii) quasi-irreversible binders (also referred to as suicide inhibitors) in which the inhibitors interact directly with the heme-iron; iii) mechanism-based inhibitors, which irreversibly bind to the protein and accelerate degradation or oxidative fragmentation of the heme (Kalgutkar et al., 2007; Correia Ortiz de Montellano, 2015; Watanabe et al., 2016). Compounds which inhibit the enzyme before the oxidative events occur are usually reversible competitive or noncompetitive inhibitors. Mechanism-based or suicide inhibitors more often act upon the oxygen transfer event or subsequent to this step (Kalgutkar et al., 2007). The latter category can have severe consequences; the complete inactivation of the enzyme means that for several hours to days the plasma concentrations of drugs which depend on the enzyme activity to become metabolized will increase, which in turn increases the risk on adverse reactions (Subehan et al., 2006). Previous identified CYP2D6 mechanism-based inhibitor drugs include paroxetine, 3,4-methylenedioxymethamphetamine (MDMA), (1-[(2-ethyl-4-methyl-1H(-EMTPP-imidazol-5-yl)-methyl]-4-[4-(trifluoromethyl)-2-pyridinyl]piperazine (EMTPP), and 5-fluoro-2-[4-[(2-phenyl-1H-imidazol-5-yl)methyl]-1-piperazinyl]pyrimidine (SCH66712) (Sridar et al., 2002; Hollenberg et al., 2008; Livezey et al., 2012). Also compounds from several natural sources such as St. John’s wort, common sage, and goldenseal/berberine have been identified to exercise a moderate to strong inhibition effect on CYP2D6 wild-type (WT) and also on allelic variants (e.g. CYP2D6*10) (Hellum and Nilsen, 2007; Gurley et al., 2008; Qu et al., 2014). Hence there is an urgent need for cost- and time-efficient methods which can help to determine the safety profile not only of (new) drugs but also of natural compounds with regard to their potential to inhibit CYP2D6 WT and clinically relevant allelic variants. An advantageous combination of methods used for identification of herbal compounds that potentially inhibit CYP2D6 consists of an *in silico* approach for a first fast screening, whereas resulting hits can be further verified by experimental *in vitro/in vivo* studies (Hochleitner et al., 2017; Vijayakumar et al., 2017). Recently, Hochleiter and co-workers successfully demonstrated the potential and relevance of a combined *in silico*–*in vitro* based workflow (including pharmacophore screening and docking) to identify new compounds derived from a natural source that inhibited CYP2D6 WT (Hochleitner et al., 2017).

In this *in silico* study the safety profile of several compounds from herbs which are known or suggested to have antidepressant activity are assessed by: (i) determining their inhibitory effect on CYP2D6 WT and the clinical relevant CYP2D6*53 (F120I, A122S) allelic variant (Sakuyama et al., 2008) and (ii) assessing their physiochemical properties along with several toxicity-related descriptors in order to define their brain bioavailability potential and off-target binding. The CYP2D6*53 allelic variant has been only identified among the Japanese with an allele frequency of 0.2% (Sakuyama et al., 2008). Moreover, it is the only allelic variant associated with increased metabolism activity (fourfold increase in CL_int_ value) for the typical CYP2D6 substrate bufuralol. The increased activity is suggested to be caused by the F120I mutation which is positioned close to the heme. Therefore, investigating and comparing the docking poses of the WT and CYP2D6*53 can provide insight regarding the role of the F120I mutation. The safety profiles compiled for each herbal compound will give a first indication if any toxicity issues might arise when they are used to treat depression, especially in multidrug therapy. It has to be mentioned that the focus of this study is on herbal compounds which are drug-like in terms of size and physiochemical properties. The herbal compounds which were not included in the analysis because they did not comply with the library filtering criteria applied, might still be suitable as antidepressant as they might cross the BBB and modulate the CNS system using any of the active transport mechanisms. However, evaluating their safety profile is much more complex and this falls outside the scope of our study.

## Materials and Methods

### Compound Library Selection

The anti-depressant natural compound library was obtained from ChemFaces (accessed July 2019), a high-purity natural products manufacturer (Wuhan, P.R. China). The 3D coordinate files were obtained from ChemSpider. For the 19 out of 51 compounds which passed filtering, a literature search was performed using PubMed and Scopus to verify their suggested antidepressant activity.

### CYP2D6 Structure Selection

For the available co-crystalized structures [Protein Data bank (PDB) IDs: 3QM4, 3TDA, 4WNT, 4WNU, 4WNV, 4WNW, 3TBG] it has been shown that the all-atom root-mean-square deviation (RMSD) of the superimposed binding sites is lower than 1 Å (Martiny et al., 2015). A major motivation of this study is to help avoiding the CYP2D6 toxicity within a co-therapy context, hence competitive binding. At the moment, there is only one protein-ligand complex structure available with a co-crystalized substrate molecule: thioridazine (a typical antipsychotic drug, PDB ID: 4WNW). Therefore, 4WNW was primarily used for docking and the binding mode analysis of the herbal compounds. However, two additional structures (PDB IDs: 4WNT and 4WNU), co-crystalized with the natural inhibitors ajmalicine and quinidine respectively, were used as well in order to verify consensus of the most favorably scored binding mode.

### Library and Protein Preparation

The structures were processed using the standardized ligand preparation procedure as implemented in the software LigPrep (Schrödinger LLC.) The preparation procedure included bond order assessment, tautomeric state, protonation evaluation (at pH 7.4), and chemical structure consistency checks. For all the compounds, the program MacroModel was used for the minimization of the starting geometries. The three selected CYP2D6 crystal structures (PDB IDs: 4WNW, 4WNU, and 4WNT, chain A) were retrieved from the PDB database and processed using the Protein Preparation Wizard of Maestro small-molecule drug discovery suite (v. 2017-2). Missing residues and hydrogen atoms were added (at pH 7.4), bond orders were assigned, and the co-crystalized ligand and co-factors were removed. No crystal waters are resolved in 4WNW and the crystal waters in 4WNU and 4WNT were deleted. Compound state I was modeled (Fe^3+^ bound to O^2−^, zero-order bond). The distance between the iron and the oxygen atoms was set to 1.97 Å. For the allelic variant CYP2D6*53 the two residues of the wild type structure were mutated (F120I, A122S) subsequently the mutated structure was fully minimized.

### Toxicokinetic Parameters and Descriptor Calculations

For each studied ligand, the QikProp program by Schrödinger was used to calculate the Lipinski’s rule of 5 (hydrogen bond donor and acceptor counts, molecular weight, logarithm of the partition coefficient), Veber rules (polar surface area, number of rotatable bonds), and several other absorption, distribution, metabolism, and excretion (ADME) and toxicity related descriptors offered by Schrödinger (log_HERG_, P_Caco_, log_BB_, P_MDCK_, Human Oral Absorption, and CNS activity score). The models behind the calculations assume absorption through passive permeation. The descriptions can be found in **Table S6**. Most psychoactive (CNS) drugs need to cross the BBB for the modulation of a target neurotransmitter system (He et al., 2018). Improved BBB permeation is usually obtained for such drugs by following narrower Lipinski ranges compared to other drugs (Pajouhesh and Lenz, 2005; Ghose et al., 2011). Two prominent CNS studies which defined the pharmacokinetic properties (including Lipinski’s rules) were used as filtering guideline and an overview of them can be found in **Table 1**. In addition, the online tool Molinspiration (www.molinspiration.com/cgi-bin/properties) was used to assess structural similarity and thus potential biological activity toward six important drug classes (G-protein coupled receptor (GPCR) ligands, ion channel modulators, kinase inhibitors, nuclear receptor ligands, protease inhibitors, enzyme inhibitors).

**Table 1:** Guidelines for the physicochemical properties associated with improved blood-brain-barrier (BBB) penetration for central nervous system (CNS) drug design.

	Ghose^a^		Pajouhesh^b^	Wager^c^	Lipinski	
Descriptor	min	max		(max)	CNS^d^	non-CNS
**MW**	141	452	<450	≤360 (500)	≤400	<500
**HbD**	0	3	<3	≤0.5 (3.5)	≤3	<5
**HbA**	1	8	<7	–	≤7	<10
**logP_o/w_**	0.16	6	<5	≤3 (5)	≤5	<5
**logS**	−0.4	0.5	–	–	–	–
**PSA**	3.8	109	60 - 70	40 – 90 (120)	–	<140
**rotB**	0	8	<8	–	–	<10

^a^based on 317 approved CNS drugs, ^b^based on consensus of several studies discussed in the paper, ^c^derived from a set of 1500 drugs filtered from United States Adopted Names (USAN) or International Nonproprietary names (INN) for good CNS penetration by Lipinski. Descriptor meaning: MW, molecular weight; HbD, number of H-bond donors; HbA, number of H-bond acceptors; logP_o/w_, logarithm of the oil/water partition coefficient; logS, logarithm of the solubility coefficient; PSA, polar surface area of the molecule; rotB, number of rotatable bonds in the molecule.

### Autodock Smina Docking

Smina, a fork of Autodock Vina (v.1.1.2) was used for docking. Smina is focused on improving scoring and minimization and includes several convenient functions which can be accessed from the command line such as calculating the box dimensions based on one or several existing ligand(s) (Koes et al., 2013). The prepared library and CYP2D6 prepared protein structures (WT and CYP2D6*53) were used for the docking. The residues F112, F120 (or I120 in the variant), E211, E215, E216, R221, Q244, R296, I297, D301, S304, and F483 of CYP2D6 were defined as flexible residues (side chains) during the docking. The random seed number was set to 0 and an additional 8 Å buffer space was added to the auto-generated box. All other settings were kept at their default values.

## Results

### Pharmacokinetics Descriptor Based Filtering

**Figure 1:**
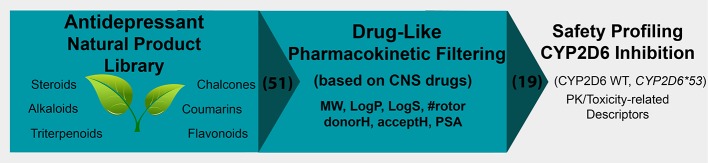
The *in silico* safety profiling approach of the natural product antidepressant library.

The ChemFaces antidepressant compound library contained 51 molecules for which all pharmacokinetic descriptors were calculated using the program QikProp (Schrödinger LLC.). Filtering criteria were based on pharmacokinetic property guidelines specific for CNS therapeutic agents (**Figure 1**). Earlier CNS pharmacokinetics analysis studies demonstrated that CNS drugs fit within a smaller range (**Table 1**—Lipinski non-CNS column) compared to the general Lipinski guidelines (**Table 1**—Lipinski CNS column) (Pajouhesh and Lenz, 2005).

Pajouhesh et al. provided guidelines for CNS drugs based on the consensus of several studies discussed in their paper (Pajouhesh and Lenz, 2005). Other CNS focused physicochemical analyses found similar reference values with some variation (**Table 1**). Ghose et al. calculated for 317 CNS and 627 non-CNS approved drugs the corresponding physicochemical properties and the preferred ranges including several pharmacokinetic descriptors using QikProp (Ghose et al., 2011). Wager et al. analyzed 119 CNS drugs and 108 Pfizer CNS candidates (Wager et al., 2010). An overview of all proposed CNS guidelines can be found in **Table 1**. For filtering of the compounds, the consensus values of Pajouhesh were applied as guidelines. In addition, if one of the descriptors was lying outside the guideline range, the other proposed ranges (Ghose or Wagner, **Table 1**) were used to validate if the value was still acceptable. Compounds with one violations of the Pajouhesh guidelines are highlighted in **Table 2**. It must be mentioned that the compounds which do not comply with the used filter criteria might still be able to cross the BBB and modulate the CNS system through an active transport.

**Table 2:** Pharmacokinetic properties of the natural antidepressants after filtering for drug-like properties.

Compound	MW	HbD	HbA	logP_o/w_	logS	PSA	rotB
(−)-Cytisine	190	1	5	0.7	−0.8	45	0
**4-Hydroxyisoleucine**	147	4	5	−2.5	0.0	91	5
5-Isopropyl-2 methylphenol	150	1	1	3.3	−2.3	21	2
Auraptenol	260	1	5	2.2	−2.6	61	5
Chelidonic acid	184	2	7	−0.6	−1.0	140	2
D-(−)-Synephrine	167	3	4	0.2	−0.3	58	5
Honokiol	266	2	2	5.0	−4.3	42	7
**Isorhynchophylline**	385	1	8	2.6	−4.0	85	4
**L-Theanine**	174	4	6	−3.0	0.2	111	6
Magnolol	266	2	2	5.0	−4.2	42	7
**Naringenin**	272	2	4	1.6	−3.4	100	3
Orcinol	124	2	2	0.8	0.1	45	2
Piperine	285	0	5	3.3	−3.5	48	5
Protopine	353	0	7	1.7	−1.1	60	0
Psoralidin	336	2	5	3.0	−5.0	92	4
Salvigenin	328	0	5	3.3	−4.1	77	4
Scopoletin	192	1	4	0.8	−1.7	71	2
Trans-methylisoeugenol	178	0	2	2.8	−3.7	16	3
**Cannabidiol (CBC)**	315	2	2	5.3	−6.0	39	7

The bold highlighted compounds have one or two violation(s) of the Pajouhesh CNS guidelines of which at least one is still within the acceptable regions guidelines of Ghose.

Descriptor meaning: MW, molecular weight; HbD, number of H-bond donors; HbA, number of H-bond acceptors; logP_o/w_, logarithm of the oil/water partition coefficient; logS, logarithm of the solubility coefficient; PSA, polar surface area of the molecule; rotB, number of rotatable bonds in the molecule.

**Figure 2:**
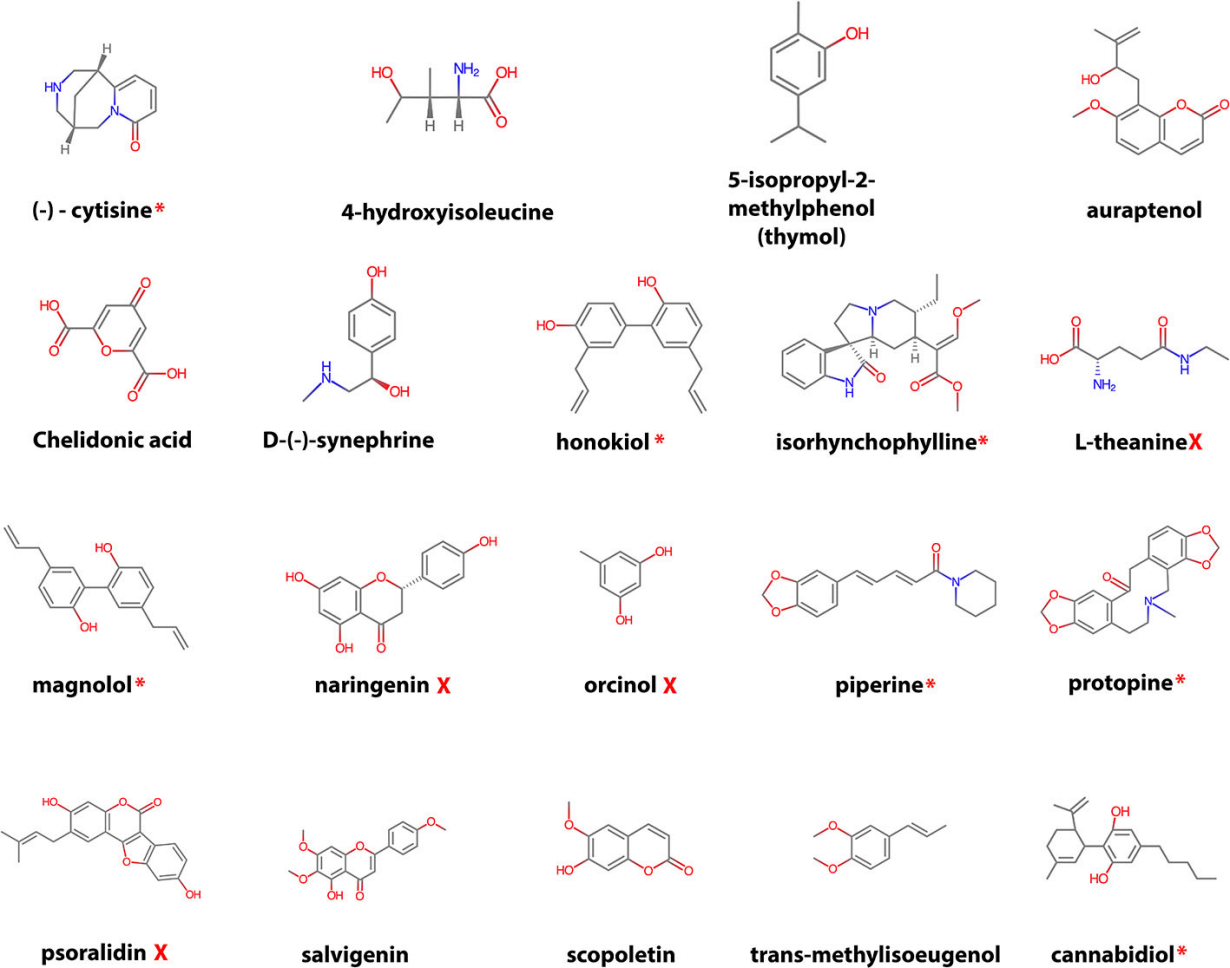
Overview of the selected natural compounds. The red asterisk indicates that previous research found CYP2D6 inhibition (potent to weak) activity for the compound (see **Table S1** for the reference), the red cross no CYP2D6 inhibition activity. For the remaining compounds no CYP2D6 inhibition data could be found.

After filtering, the remaining 19 compounds (**Figure 2**) were docked in CYP2D6 WT and the CYP2D6*53 allelic variant for the three prepared CYP2D6 structures (PDB IDs: 4WNW, 4WNU, and 4WNT) and several pharmacokinetic descriptors were calculated. In addition, the literature was searched if any evidence existed on CYP2D6 inhibition (**Table S1**), P450 (major) isoform metabolism (**Table S2**), and their ability to cross the BBB (**Table S3**).

### CYP2D6 Inhibition

CYP2D6 WT inhibition activity (varying from weak to potent inhibition) data was found for seven compounds, four compounds were identified to have no inhibition activity, and for eight no data could be found (**Table S1**). Though the neolignans honokiol and magnolol present in magnolia bark extract have been considered to be safe in use by various authorities (Sarrica et al., 2018), evidence exists on CYP2D6 inhibition for magnolol (IC_50_ 65.4 µM) (Zhang et al., 2019) and weak inhibition for honokiol (Ki 12 µM) (Jeong et al., 2013). Cannabidiol (CBD) one of the three major components in the cannabis plant has been identified as potent atypical inhibitor (IC_50_ 6.52 µM) of CYP2D6 (Yamaori et al., 2011). Furthermore, for piperine weak inhibition (IC_50_ 3.2 µM) and protopine potent competitive inhibition (Ki 78 nM) has been determined (Li et al., 2011; Shamsi et al., 2017). Moderate CYP2D6 WT inhibition was found for (−)-cytisine (IC_50_ 28.9 µmol/L) and isorhynchophylline (IC_50_ 44.1 µmol/L) (Qu et al., 2014). Literature search confirmed for 13 compounds that they are able to cross the BBB (**Table S3**). Whether they reach a high enough concentration to acquire and maintain their bioactivity within the brain remains an open question and needs to be confirmed by additional clinical studies. Experimental evidence of CYP-mediated metabolism was found for nine of the compounds, one has been assigned no CYP-metabolism dependence, and for other nine no data was found (**Table S2**). No literature was found that assigned CYP2D6 as major isoform for metabolism for one of the compounds.

### Inhibition Binding Modes From Molecular Docking

The top 10 binding poses were evaluated on potential CYP2D6 inhibition. The distance of the binding pose (distal or proximal) between the heme and the ligand was evaluated as well as the functional group and closest atom to the iron heme. If the distance between the heme-iron and any atom of the ligand was below 6 Å it was assigned proximal binding, otherwise it was assigned as distal. Several residues in the binding pocket are essential for ligand binding. Glu216 and/or Asp301 are known to act as first anchoring point by forming salt bridges with the commonly protonated aliphatic nitrogen atom (at physical pH) present in most typical CYP2D6 substrates. Subsequently Phe120 which resides in close proximity of the heme can further steer the orientation of the ligand by interacting with the aromatic part of the substrate through π-π stacking interactions (Rowland et al., 2006; Wang et al., 2009; Wang et al., 2015). An example of inhibition can be observed in the crystal CYP2D6 structure (PDB ID: 4WNU) where quinidine (inhibitor) binds distal from the heme and its protonated nitrogen forms electrostatic interactions with both E216 and D301 (Wang et al., 2015). Based on this knowledge docking binding modes were assigned to potentially inhibit CYP2D6 if (i) a functional group (known not to be normally metabolized) was interacting directly with the iron heme and (ii) the distal binding from the heme prevented other ligands to reach the heme and/or blocked interaction with D301 and/or E216. Furthermore, the binding profile of the pose was evaluated on the presence of potential electrostatic interactions, hydrogen bonding, and hydrophobic interactions. The docking results can be found in **Table 3** and a detailed overview of the different interactions types of the best binding modes can be found in **Tables S4** and **S5**.

**Table 3:** Overview CYP2D6 WT and CYP2D6*53 docking results.

	CYP2D6 wild-type			CYP2D6*53		
**Compounds**	**prox**	**dis**	**none**	**prox**	**dis**	**none**
(−**)-Cytisine**						
**4-Hydroxyisoleucine**						
**5-Isopropyl-2-methylphenol**						
**(S)-Auraptenol**						
**Chelidonic acid**						
**D-(**−**)-synephrine**						
**Honokiol**						
**Isorhynchopylline**						
**L-theanine**						
**Magnolol**						
**Naringenin**						
**Orcinol**						
**Piperine**						
**Protopine**						
**Psoralidin**						
**Salvigenin**						
**Scopoletin**						
**Trans-methylisoeugenol**						
**Cannabidiol (CBD)**						

The top 10 docking poses were evaluated on potential CYP2D6 inhibition by evaluating its binding distance toward the heme (proximal or distal) together with its binding pocket interaction profile.

**Figure 3:**
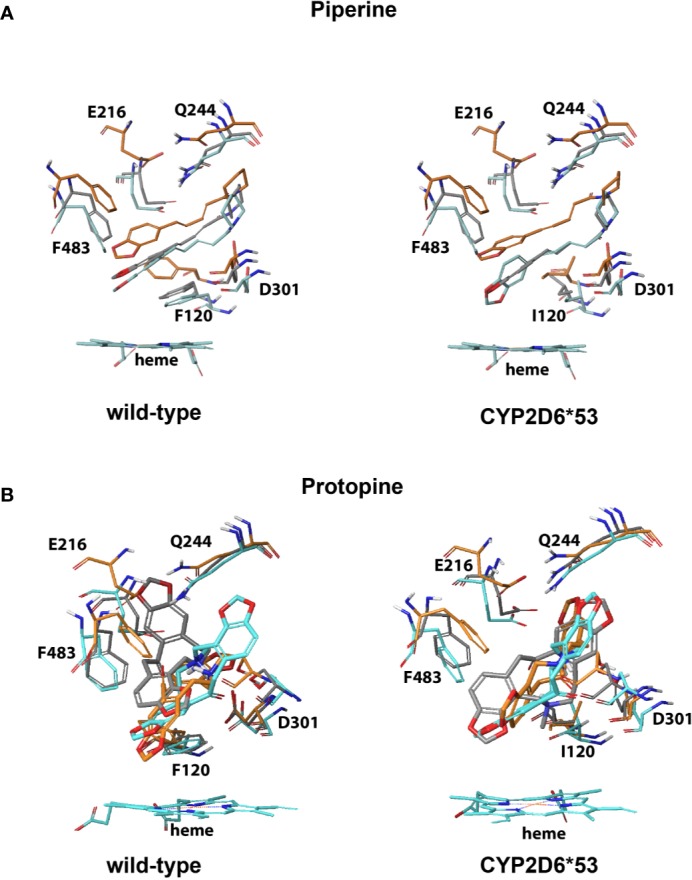
CYP2D6 WT and CYP2D6*53 inhibition binding modes for **(A)** piperine and **(B)** protopine. The best scored poses are shown docked into 4WNW (cyan), 4WNU (orange), and 4WNT (grey). To keep a clear overview, only the interacting residues are displayed and labeled and solely the heme of 4WNW is displayed.

Within the top 10, the most productive pose(s) (based on the mentioned criteria above) proximal and/or distal from the heme were selected. An interaction profile for the herbal compounds that were binding (proximal and/or distal) can be found in the supplementary data (**Figures S1–S3**). From the seven compounds for which experimental evidence was found on CYP2D6 WT inhibition activity, the binding poses of piperine, protopine, honokiol, magnolol, and CBD were found to potentially inhibit CYP2D6 WT and CYP2D6*53; the binding mode of piperine in the WT was more strongly stabilized by hydrophobic interactions with F120 than with I120 in CYP2D6*53 (**Figures 3** and **5B**). Protopine formed mainly electrostatic/hydrophobic interactions with the heme group. In CYP2D6 WT π-π stacking was observed with F483 whereas a hydrogen bond with Q244 was formed in CYP2D6*53 (**Figure 3**).

**Figure 4:**
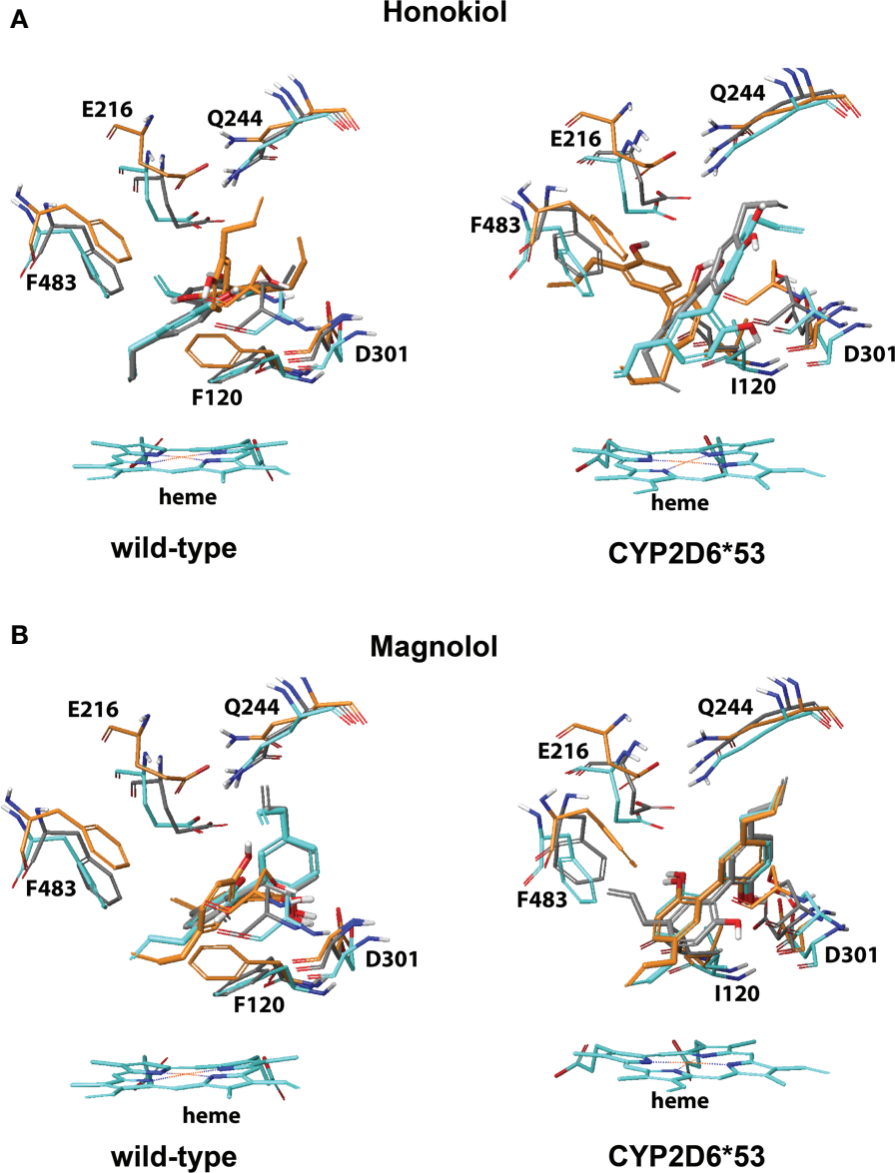
CYP2D6 wild-type and CYP2D6*53 inhibition binding modes for **(A)** Honokiol and **(B)** Magnolol. The best scored poses are shown docked into 4WNW (cyan), 4WNU (orange) and 4WNT (grey). To keep a clear overview, only the interacting residues are displayed and labeled and solely the heme of 4WNW is displayed.

The binding modes observed (proximal and also distal) for honokiol and magnolol in CYP2D6 WT and CYP2D6*53 were such that one of the two ethylene tails pointed toward the heme and one of the hydroxyl groups formed electrostatic interactions with either D301 or a stabilizing hydrogen bond with S304 (**Figure 4** and **Figure S3**). In addition, for CYP2D6 WT, hydrophobic interactions with F120 and one of the phenyl rings was observed.

For CBD, the best scored binding modes pointed the aliphatic pentyl tail toward the heme and one of the two hydroxyl groups was stabilized by either a hydrogen bond (S304, Q244) or electrostatic interaction with D301 in CYP2D6 WT and CYP2D6*53 (**Figure S2**). In CYP2D6 WT stabilizing hydrophobic interactions between F120 and the aromatic ring were found for honokiol and magnolol (**Figure S3**). For cytisine, a binding pose pointing toward potential inhibition was only observed in the CYP2D6 WT; the protonated nitrogen of cytisine formed an electrostatic interaction with D301. For isorhynchophylline no reasonable binding poses that could explain its inhibitory effect on CYP2D6 were generated.

The compounds L-theanine, orcinol, naringenin, and psoralidin for which experimental studies confirmed no CYP2D6 WT inhibition (**Figure 2**, **Table S1**), docking results did neither show any decisive inhibition binding modes in CYP2D6 WT or CYP2D6*53. For the remaining eight compounds for which no experimental CYP2D6 inhibition data was found, 4-hydroxyisoleucine, chelidonic acid, salvigenin, and trans-methylisoeugenol did not show any CYP2D6 inhibition binding modes in both CYP2D6 WT or CYP2D6*53. The proximal binding poses of thymol (5-isopropyl-2-methylphenol) formed hydrophobic interactions with F120 and hydrophobic/electrostatic interactions with the heme group in CYP2D6 WT (**Figure S1**).

For the distal binding modes of (S)-auraptenol, the hydroxyl group formed a hydrogen bond with S304 or/and also electrostatic interactions with D301 or E216 in CYP2D6 WT or CYP2D6*53 (**Figure S1**). In addition, π-π stacking between the hydrophobic ring and F120 was observed for the highest ranked pose in CYP2D6 WT. For D-(−)-synephrine proximal binding was found in CYP2D6 WT with mainly electrostatic stabilizing interactions (E216 and Q244) whereas proximal binding in CYP2D6*53 with the protonated nitrogen forming electrostatic interactions with D301 (**Figure S2**). Scopoletin showed a similar proximal binding mode in which the oxygen pointed in the direction of the heme group in both CYP2D6 WT and CYP2D6*53 (**Figure S2**).

### Pharmacokinetic and Toxicity-Related Descriptors

Several pharmacokinetic descriptors calculated with QikProp can be found in **Tables 4** **and** **5**. Chelidonic acid, L-theanine, and naringenin have all a PSA above 100 Å, and 4-hydroxyisoleucine and isorhynchophylline exceed the number of acceptable hydrogen bond donors or acceptors for CNS drugs which could decrease their ability to permeate the BBB (**Table 2**), although experimentally evidence for all except chelidonic acid exists that they cross the BBB (**Table S3**). From the toxicity-related calculated QikProp descriptors (**Table 4**), eight compounds have one or more value(s) lying outside the recommended regions. 4-Hydroxyisoleucine, chelidonic acid, and L-theanine have P_caco_ and P_MDCK_ predicted values indicating potential issues with cell permeability, which is also indicated by their predicted low human oral absorption and inactive (below 0) CNS score. Naringenin and psoralidin are also predicted to be CNS inactive. CBD is a known inhibitor of the P-glycoprotein, which substantially affects its bioavailability (Zhu et al., 2006). Honokiol, magnolol, isorhynchophylline, psoralidin, and salvigenin are predicted to interact with the hERG channel. Furthermore, the online prediction platform Molinspiration (www.molinspiration.com) was used for prediction of a bioactivity score toward six of the most important drug classes (GPCRs, ion channel modulators, kinase inhibitors, nuclear receptor ligands, protease inhibitors, enzyme inhibitors) based on the structure similarity with typical class representatives (**Table 5**).

**Table 4:** QikProp calculated toxicity-related descriptors.

Compound	log_HERG_	P_Caco_ (nm/s)	logBB	P_MDCK_ (nm/s)	HOA (%)	CNS
(−)-Cytisine	−3.8	491	0.4	254	79	1
**4-Hydroxyisoleucine**	−1.5	**15**	−0.6	**8**	34	−**1**
5-Isopropyl-2-methylphenol	−3.6	3697	0.1	2033	100	1
Auraptenol	−4.0	1323	−0.5	669	96	0
**Chelidonic acid**	0.3	**1**	−1.8	1	**26**	−**2**
D-(−)-synephrine	−4.8	193	−0.3	92	69	0
**Honokiol**	−**5.8**	1615	−0.7	830	100	0
**Isorhynchophylline**	−**6.1**	263	−0.3	129	86	1
**L-theanine**	−1.0	**6**	−1.1	4	**23**	−**2**
**Magnolol**	−**5.7**	1717	−0.6	887	100	0
**Naringenin**	−5.0	130	−1.4	55	74	−**2**
Orcinol	−3.3	912	−0.4	448	85	0
Piperine	−4.8	3980	−0.1	2202	100	0
Protopine	−4.4	1298	0.7	725	93	2
**Psoralidin**	−**5.7**	312	−1.3	140	89	−**2**
**Salvigenin**	−**5.2**	1504	−0.5	769	100	0
Scopoletin	−3.8	627	−0.6	299	82	0
Trans-methylisoeugenol	−3.9	9906	0.1	5899	100	1
Cannabidiol (CBD)	−3.8	491	0.4	254	79	1

A detailed description of each descriptor can be found in **Table S6**.

Descriptor meaning: log_HERG_, predicted logarithm of the IC_50_ value for blockage of HERG K+ channels; P_Caco_, predicted apparent Caco-2 cell permeability; logBB, predicted brain/blood partition coefficient; P_MDCK_, predicted apparent MDCK cell permeability coefficient; HOA, predicted human oral absorption on 0 to 100% scale; CNS, predicted central nervous system activity on a –2 (inactive) to +2 (active) scale. Unfavorable scores are highlighted in red. Bold font indicates compounds with problematic ADMET properties.

**Table 5:** Bioactivity prediction score for the 19 compounds toward six important drug classes.

Compound | Drug class	GPCR ligand	Ion channel modulator		Kinase inhibitor	Nuclear receptor ligand	Protease inhibitor	Enzyme inhibitor
(−)**-Cytisine**	−**0.58**	**0.39**	−**0.75**		−**1.1**	−**0.62**	−**0.25**
4-Hydroxyisoleucine	−0.72	−0.26	−1.31		−0.96	−0.71	−0.31
5-Isopropyl-2-methylphenol	−1.02	−0.51	−1.15		−0.7	−1.25	−0.56
Auraptenol	−0.24	−0.56	−0.73		0.14	−0.56	0.11
Chelidonic acid	−1.05	−0.56	−0.93		−1.02	−0.92	−0.4
D-(−)-Synephrine	−0.39	0.07	−0.79		−0.51	−0.88	−0.04
**Honokiol**	**0.04**	**0.06**	−**0.08**		**0.32**	−**0.2**	**0.13**
**Isorhynchophylline**	**0.26**	**0.19**	−**0.3**		**0.03**	−**0.22**	**0.01**
L-Theanine	−0.53	−0.15	−1.15		−1.42	−0.08	−0.4
Magnolol	−0.01	0.05	−0.15		0.2	−0.23	0.07
**Naringenin**	**0.03**	−**0.2**	−**0.26**		**0.42**	−**0.12**	**0.21**
Orcinol	−2.26	−1.64	−2.35		−2.1	−2.59	−1.77
Piperine	0.15	−0.18	−0.13		−0.13	−0.1	0.04
**Protopine**	**0.2**	**0.07**	−**0.35**		−**0.24**	−**0.07**	**0.17**
**Psoralidin**	−**0.2**	−**0.09**	−**0.17**		**0.53**	−**0.15**	**0.21**
Salvigenin	−0.11	−0.27	0.15		0.13	−0.29	0.11
Scopoletin	−1	−0.65	−0.95		−0.81	−1.16	−0.24
Trans-Methylisoeugenol	−0.95	−0.53	−0.98		−0.72	−1.2	−0.53
**Cannabidiol (CBD)**	**0.35**	−**0.14**	−**0.48**		**0.38**	−**0.19**	**0.33**

Favorable predicted scores are highlighted yellow, unfavorable red. Bold text indicates compounds with significant predicted bioactivities.

A bioactivity prediction score above 0 indicates a high similarity to existing active compound. Considering the fact that GPCRs are paramount for regulation of mood, pain, cognition, and neurotransmitter release through synaptic transmission, predicted bioactivity toward those receptors would be expected for the modeled compounds (Huang and Thathiah, 2015). Several CNS drugs (e.g. tricyclics such as amitriptyline) modulate ion channel activity to reduce depression in human (Kaczorowski et al., 2008; Lodge and Li, 2008) hence a bioactivity score for ion channel modulation above 0 would be considered as a positive result. For the remaining four drug classes (kinase inhibitors, nuclear receptor ligands, protease inhibitors, enzyme inhibitors) no similarity (activity) is desired. **Table 5** shows that cytisine, isorhynchophylline, naringenin, protopine, and CBD are predicted to be similar (active) toward the GPCR ligand and/or the ion channel modulator drug class. Honokiol, naringenin, psoralidin, and CBD are predicted to be similar (active) toward one of the four undesired drug classes.

## Discussion

In this study the safety profile of 19 natural compounds was evaluated on CYP2D6 inhibition using an *in silico* approach. Inhibition was investigated for CYP2D6 WT and the allelic variant CYP2D6*53 which is associated with increased metabolism activity for bufuralol. The variant contains two amino acid mutations (F120I, A122S) of which F120I is positioned in close vicinity of the heme. Comparison of the docking binding poses of the WT and variant can provide insight on the function of the mutation in modulating the accessibility of the compound to the heme. In addition, it may give an indication regarding the impact of allelic variants with amino acid mutations close to the heme on inducing inhibition. Moreover, it is generally accepted that accurate site of metabolism prediction requires to probe the accessibility and the reactivity of the ligand atoms (Tyzack and Kirchmair, 2019). However, for the aim of this study in which the focus is on inhibition, using a time-efficient structure-based method such as docking to investigate the compound-CYP interactions and subsequently deduce its inhibition potential has previously shown to be a valuable pre-screening strategy (Martiny et al., 2015, 6; Hochleitner et al., 2017; Dutkiewicz and Mikstacka, 2018). Subsequently, *in vitro* and *in vivo* safety studies can be performed for examination of the smaller compound set. The results from the natural compound docking correlated six out of seven experimentally confirmed CYP2D6 WT inhibitors; honokiol, magnolol, CBD, piperine, protopine, and cytisine. In the allelic variant CYP2D6*53, potent inhibition binding modes were observed as well except for cytisine. The docking poses of (S)-auraptenol and scopoletin in CYP2D6 WT and CYP2D6*53 pointed toward potential inhibition activity as well. 5-Isopropyl-2-methylphenol formed electrostatic interactions with the prosthetic heme group in CYP2D6 WT only. The mutation F120I enabled for some compounds [especially D-(−)-synephrine, scopoletin, (−)-cytisine] to get closer to the heme-oxygen. These compounds have in common that they are relatively small and less flexible, which restricts their possible binding modes to the heme-oxygen compared to larger more flexible compounds such as for example CBD or magnolol.

**Figure 5:**
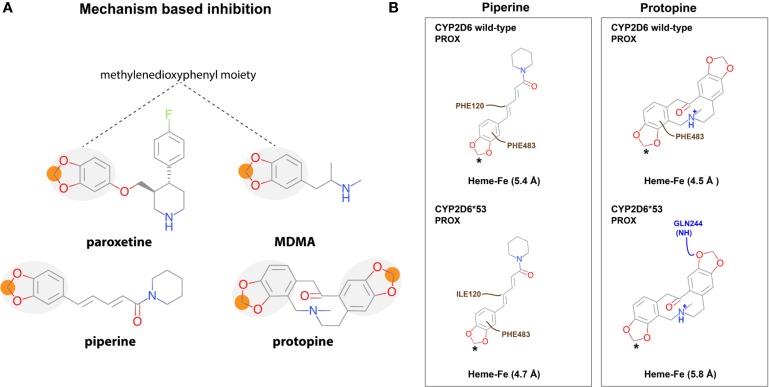
Mechanism based CYP2D6 inhibition. **(A)** Piperine and protopine both contain a methylenedioxyphenyl moiety which has been associated with mechanism-based inhibition (MBI) of CYP2D6 (e.g. paroxetine and MDMA are CYP2D6 MBI). **(B)** The best scored binding mode (in 2D) for each is shown in CYP2D6 WT and CYP2D6*53. The asterisk indicates the atom closest to the heme-iron. Color code: hydrophobic interactions; brown, electrostatics; purple, and hydrogen bonds; blue.

An interesting observation was made for the alkaloids piperine and protopine both containing a methylenedioxyphenyl moiety (**Figure 5A**). The highest scored docking pose of piperine and protopine in both CYP2D6 WT and CYP2D6*53 positioned the moiety in proximal vicinity of the heme (**Figure 5B** **and** **Figure 3**).

As described in the introduction, identification of mechanism-based inhibitors (MBI) is particularly relevant considering the fact that they bind irreversible and thus completely inactivate the enzyme. Subsequently, during the time that the body needs to produce new CYP2D6 enzymes which can take hours to days (e.g. 70 h in case of paroxetine) the plasma concentration of drugs which require CYP2D6 activity for their biotransformation will increase which in turn increases the chance on adverse reactions (Liston et al., 2002). Previously identified CYP2D6 MBI drugs include paroxetine and MDMA (3,4-methylenedioxymethamphetamine, ecstasy) (**Figure 3**) (Sridar et al., 2002; Hollenberg et al., 2008; Livezey et al., 2012). A common feature of the drugs is their methylenedioxyphenyl moiety, which can be oxidized by CYP2D6, forming a carbene intermediate. This intermediate subsequently binds to the heme iron as an axial ligand. This knowledge may explain the experimentally determined inhibition activity of both natural compounds (Li et al., 2011; Shamsi et al., 2017) and is in accordance with the observed binding modes. However, the presence of the methylenedioxyphenyl can also lead to reversible inhibition as shown in a recent study investigating MDMA metabolism by CYP2D6 WT (Rodgers et al., 2018). Therefore, a compound containing a methylenedioxyphenyl moiety should be carefully investigated on CYP2D6 inhibition, depending on the overall structure of the compound, the type of binding can be reversible or irreversible.

The dynamic development of docking methods in recent years and their wide application in structure-based design projects especially in the pharmaceutical industry, the docking binding modes are nowadays routinely employed to assess the likelihood if a small molecule might bind at protein binding site (Ferreira et al., 2015; Ballante, 2018; Śledź and Caflisch, 2018). The accuracy of the poses and the estimation of the binding free energy however depend directly on the protocol used and may differ from target to target (Cournia et al., 2017; Mobley and Gilson, 2017; Koukos et al., 2019). As cytochrome P450s are known for their rather high flexibility our study used a flexible docking protocol. All compounds could be successfully accommodated within the binding site with reasonable binding modes allowing interpretation of the structure-activity relationships at both CYP2D6 WT and the CYP2D6*53 variant (**Figures 3** and **4**). The stability of the predicted binding modes can be further evaluated by replicated molecular dynamics (MD) simulations. This would provide additional insight into the dynamical stability of the intermolecular interactions identified within the docked binding modes. Moreover, for the cases where the binding is in close vicinity of the heme, the ultimate answer to the question if an oxidation reaction may occur at a particular ligand site can be derived from quantum mechanics/molecular mechanics (QM/MM) calculations (Friesner and Guallar, 2005; Olah et al., 2011; Reinhard and de Visser, 2017). However, both MD and QM/MM simulations belong to the most CPU-intensive approaches and are not practicable for our rather large set of compounds.

*In silico* evaluation of toxico/pharmacokinetic descriptors (**Table 4**) demonstrated that the herbal origin studied compounds generally fulfill all criteria needed for absorption from the gastrointestinal tract after oral intake. Most descriptor values fit also into the narrower range defined for CNS active drugs. Chelidonic acid, L-theanine, and naringenin feature slightly higher PSA values when calculated using 3D approach (QikProp), but cannot be directly disqualified as unpermeable; 2D based algorithms generate values closer to the upper threshold value for the CNS availability (Shityakov et al., 2013). Moreover, the PSA descriptor bears the risk of overinterpretation. For instance a hydroxyl (OH) group is more expensive to desolvate and transfer through the membrane than a carbonyl oxygen (=O), while they both contribute to the PSA with very similar fraction (20 Å vs. 17 Å^2^). Small deviations in one descriptor should not translate to complete loss of bioavailability. In fact, sufficient oral availability (> 69%) was confirmed using specialized predictive models of QikProp for 16 compounds, with remaining three being at least partially absorbed (23%–34%). Estimated permeation coefficients in the Caco-2 cells follow this trend. The two compounds, 4-hydroxyisoleucine and L-theanine, predicted to have low permeation coefficients, have an amino acid character and are therefore likely using an active transport mechanism (Smith, 2000). Descriptor values calculated using specialized modules for the CNS activity and BBB permeation showed positive scores for six compounds, eight were scored neutral and five had negative scores. Lower scores than expected for CNS active compounds might be caused by the fact that QikProp modules were trained using drug molecules, which are typically located in different region of the chemical space than natural compounds (Ioakimidis et al., 2008). The same applies for similarity based indices generated by the Molinspiration Bioactivity Predictor, which is also build on typical drug-like molecules falling into respective categories. Nevertheless, it is interesting to see that the studied compounds show similarity with different compound groups, that might be associated to their desired (antidepressant), but also undesired pharmacological effects (endocrine disruption, cardiotoxicity).

## Conclusions

Natural remedies are often associated with a safe use. Buying herbal supplements seems to be harmless as most of the time no special warnings can be found in the accompanying information. However, an increasing trend of using natural compounds as single antidepressant or in polypharmacy needs to be handled with caution. Toxicity issues may arise depending on the dose and therapy and its P450 metabolism dependence if the herbal compound or a complex product is not given safety clearance by the FDA or another established authority. CYP2D6 inhibition or off-target binding are particularly dangerous. The *in silico* results of this study indicate for several of the natural compounds suggested to be used as antidepressant that a potential increased risk may exist on adverse reactions triggered by CYP2D6 inhibition or another off-target analyzed (kinase inhibitors, nuclear receptor ligands, protease inhibitors, enzyme inhibitors, hERG binding). From the 19 natural compounds analyzed, nine indicated no CYP2D6 inhibition for both CYP2D6 WT and CYP2D6*53 (4-hydroxyisoleucine, chelidonic acid, isorhynchopylline, L-theanine, naringenin, orcinol, psoralidin, salvigenin, trans-methylisoeugenol). The other 10 natural compounds ((−)-cytisine, 5-isopropyl-2-methylphenol, s-auraptenol, D-(−)-synephrine, honokiol, magnolol, piperine, protopine, scopoletin and CBD) showed clearly potential for CYP2D6 WT and/or CYP2D6*53 inhibition. If administered with a concomitant drug which depends on CYP2D6 activity this may lead to adverse reactions. Further experimental investigations are required to confirm the outcome of the *in silico* docking, the off-target predictions and the toxicity descriptors. Especially hERG binding should be evaluated for honokiol, magnolol, isorhynchopylline, psoralidin, and salvigenin.

## Data Availability Statement

All datasets generated for this study are included in the article/**Supplementary Material**.

## Author Contributions

MS and CD designed the research. CD performed the research and analyzed the data. MS and CD wrote the manuscript.

## Conflict of Interest

The authors declare that the research was conducted in the absence of any commercial or financial relationship that could be construed as a potential conflict of interest.
