# Supplementary material for: In Silico Pharmacogenetics CYP2D6 Study Focused on the Pharmacovigilance of Herbal Antidepressants
Source: Front Pharmacol. 2020 May 13;11:683. doi: 10.3389/fphar.2020.00683 (PMC7237870; doi:10.3389/fphar.2020.00683)
Supplement: Supplementary file 1 [file DataSheet_1.pdf]

Supplementary Material

In silico pharmacogenetics CYP2D6 study focused on the pharmacovigilance of herbal antidepressants

Charleen G. Don[1#] and Martin Smieško[2*]

[1]Computational Pharmacy Group, Department of Pharmaceutical Sciences, University of Basel, Basel, Switzerland

# These authors contributed equally to this work.

*** Correspondence:**
martin.smiesko@unibas.ch

Contents

Table S1 **Overview of literature sources for CYP2D6 inhibition**

Table S2 **Overview of literature sources for P450**

Table S3 **Overview of literature sources for blood brain barrier (BBB) crossing**

Figure S1 **The best scored binding modes (in 2D) for A) (-)-Cytisine, (S)-auraptenol and 5-isopropyl-2-methylphenol and B) Scopoletin, cannabidiol (CBD) and D-(-)-synephrine are shown in CYP2D6 WT and CYP2D6*53**

Figure S2 **The best scored binding modes (in 2D) for honokiol and magnolol are shown in CYP2D6 WT and CYP2D6*53**

Table S4 **Overview of the various types of interactions found in the best binding modes for CYP2D6 WT**

Table S5 **Overview of the various types of interactions found in the best binding modes for CYP2D6*53**

Table S6 **Overview QikProp descriptors with Schrödinger explanation**

TABLE S1 | Overview of literature sources for CYP2D6 inhibition. If literature for a compound on the topic could not be found, this is indicated as “unknown”.

Natural Product	CYP2D6 inhibition	Ref
(-)-Cytisine	moderate	Qu et al., 2014
4-Hydroxyisoleucine	unknown	-
5-Isopropyl-2-methylphenol	unknown	-
Auraptenol	unknown	-
Chelidonic acid	unknown	-
D-(-)-Synephrine	unknown	-
Honokiol	moderate	Cho et al., 2014
Isorhynchophylline	moderate	Qu et al., 2014
L-Theanine	none	Sadzuka et al., 2006
Magnolol	moderate	Zhang et al., 2019
Naringenin	none	Lu et al., 2011
Orcinol	none	Yamaori et al., 2011
Piperine	weak	Shamsi et al, 2017
Protopine	potent	Li et al., 2011
Psoralidin	none	Shi et al., 2016
Salvigenin	unknown	-
Scopoletin	unknown	-
Trans-Methylisoeugenol	unknown	-
cannabidiol (CBC)	potent	Yamori et al., 2011

TABLE S2 | Overview of literature sources for P450 metabolism. If literature for a compound on the topic could not be found, this is indicated as "unknown".

**Natural Product**	**P450 metabolism (major isoforms)**	Ref
**(-)-Cytisine**	unknown	**-**
**4-Hydroxyisoleucine**	unknown	**-**
**5-Isopropyl-2-methylphenol**	CYP2A6	**Dong et al., 2012**
**Auraptenol**	unknown	**-**
**Chelidonic acid**	unknown	**-**
**D-(-)-Synephrine**	unknown	**-**
**Honokiol**	CYP1A2, CYP2C8, CYP2C9, CYP2C19	**Xu et al., 2008**
**Isorhynchophylline**	CYP2D, CYP1A1, CYP1A2, CYP2C	**Wang et al., 2010**
**L-Theanine**	none	**Sadzuka et al., 2006**
**Magnolol**	CYP2E1, CYP3A4, CYP1A2	**Huang et al., 2019**
**Naringenin**	P450-dependent	**Sousa et al., 2013**
**Orcinol**	unknown	**-**
**Piperine**	unknown	**-**
**Protopine**	CYP2D1, CYP2C11	**Paul et al., 2004**
**Psoralidin**	CYP2C19	**Shi et al., 2016**
**Salvigenin**	unknown	**-**
**Scopoletin**	unknown	**-**
**Trans-Methylisoeugenol**	P450-dependent	**Cartus et al., 2011**
**cannabidiol (CBC)**	CYP2C19, CYP3A4	**Jiang et al., 2011**

TABLE S3 | Overview of literature sources for crossing of blood brain barrier (BBB). If literature for a compound on the topic could not be found, this is indicated as "unknown".

Natural Product	BBB	Ref
**(-)-Cytisine**	poor	**Mineur et al., 2009**
**4-Hydroxyisoleucine**	unknown	**-**
**5-Isopropyl-2-methylphenol**	unknown	**-**
**Auraptenol**	unknown	**-**
**Chelidonic acid**	unknown	**-**
**D-(-)-Synephrine**	poor	**Stohs et al., 2011**
**Honokiol**	√	**Lin et al., 2012**
**Isorhynchophylline**	√	**Zhang et al., 2017**
**L-Theanine**	√	**Takeshima et al., 2016**
**Magnolol**	√	**Tsai et al., 1996**
**Naringenin**	√	**Youdim et al., 2004**
**Orcinol**	√	**Wang et al., 2015**
**Piperine**	√	**Eigenmann et al., 2016**
**Protopine**	√	**Metha et al., 2016**
**Psoralidin**	unknown	**-**
**Salvigenin**	√	**Kavvadias et al., 2004**
**Scopoletin**	√	**Basu et al., 2016**
**Trans-Methylisoeugenol**	unknown	**-**
**cannabidiol (CBC)**	√	**Blanco et al., 2016**

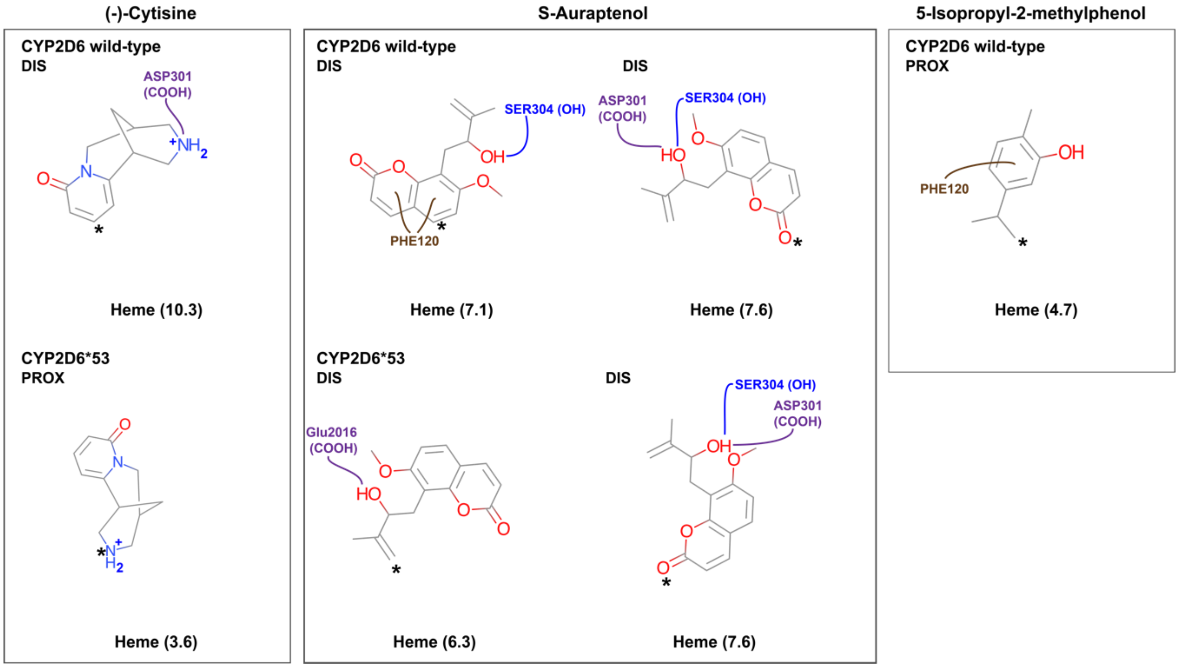

FIGURE S1 | The best scored binding modes (in 2D) for (-)-Cytisine, S-Auraptanol and 5-isopropyl-2-methylphenol are shown in CYP2D6 wild-type and CYP2D6*53. The asterisk indicates the atom closest to the heme-iron. Color code: hydrophobic interactions; brown, electrostatics; purple, and hydrogen bonds; blue. Proximal: distance between heme-Fe and closest atom < 6 Å and distal; > 6 Å.

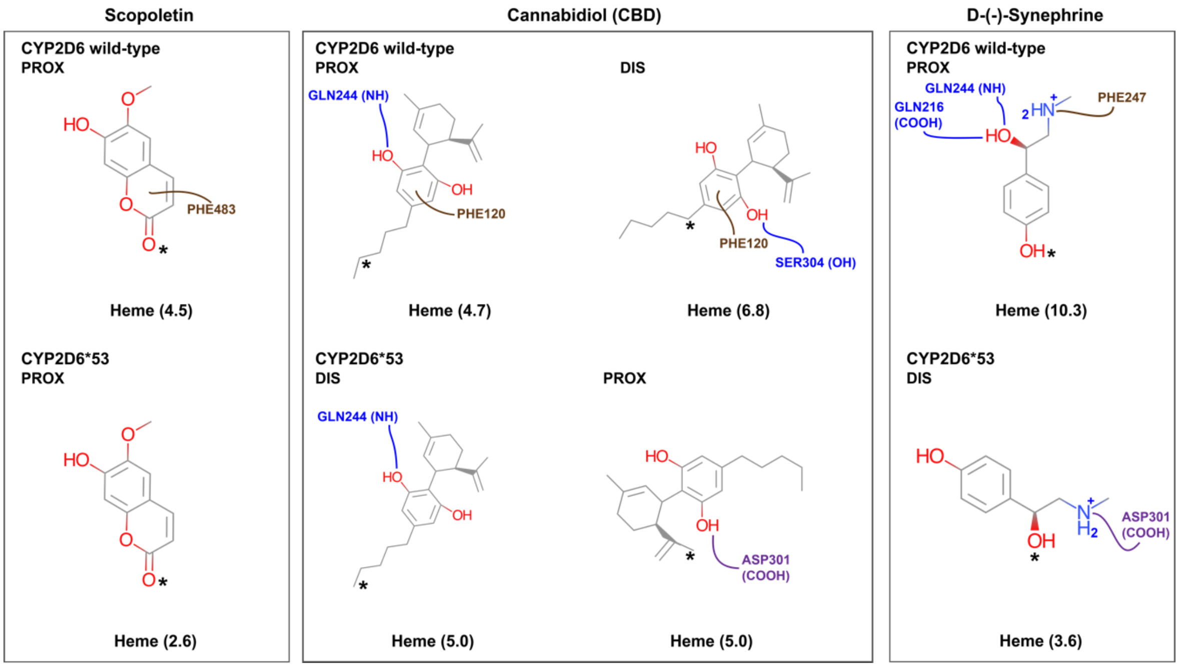

FIGURE S2 | The best scored binding modes (in 2D) for Scopoletin, Cannabidiol (CBD) and D-(-)-Synephrine are shown in CYP2D6 wild-type and CYP2D6*53. The asterisk indicates the atom closest to the heme-iron. Color code: hydrophobic interactions; brown, electrostatics; purple, and hydrogen bonds; blue. Proximal: distance between heme-Fe and closest atom < 6 Å and distal; > 6 Å.

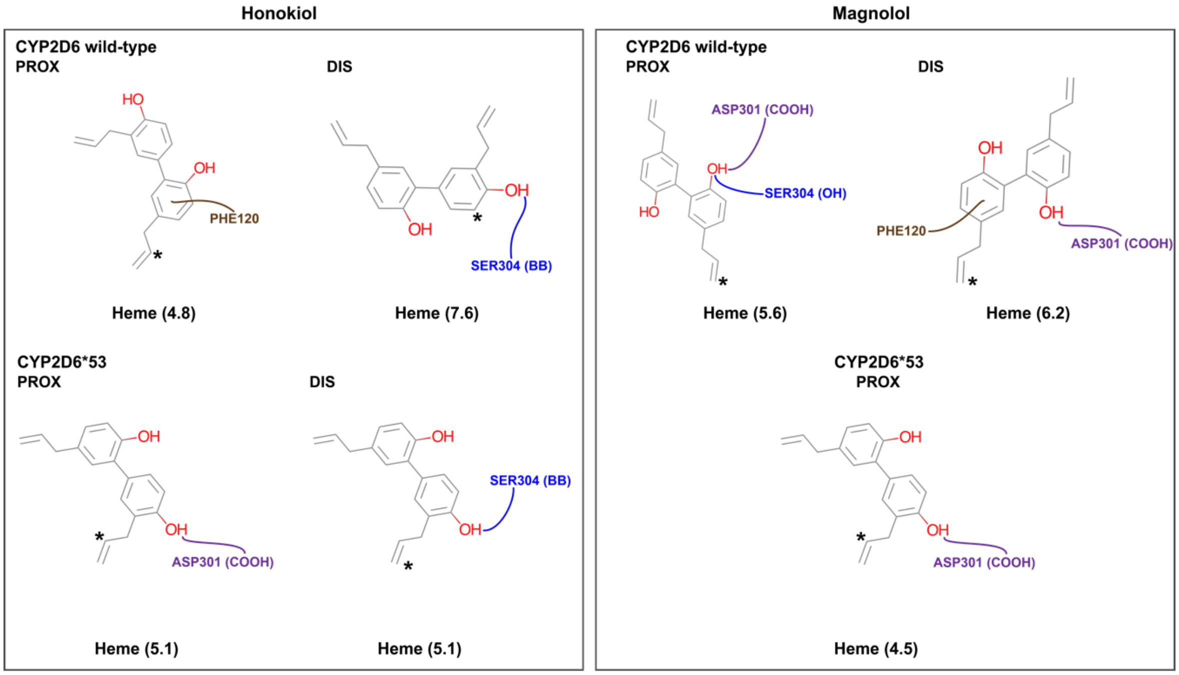

FIGURE S3 | The best scored binding modes (in 2D) for Honokiol and Magnolol are shown in CYP2D6 wild-type and CYP2D6*53. The asterisk indicates the atom closest to the heme-iron. Color code: hydrophobic interactions; brown, electrostatics; purple, and hydrogen bonds; blue. Proximal: distance between heme-Fe and closest atom < 6 Å and distal; > 6 Å.

TABLE S4 | Overview of the various types of interactions found in the best binding modes for CYP2D6 wild-type.

Natural Product		4WNW WT		Distance	Heme
		AA	Type	(Å)	(Å)
**Thymol**		F120	π stacking	~3.8	4.7
**s-auraptenol**		D301	ionic	2.3	7.6
		S304	H-bond	2.4	
**D-(-)-synephrine**	**01**	D301	ionic	2.6	6.1
		F120	π-stacking	~ 4.7	
	**02**	E216	ionic	2.3	5.1
		Q244	H-bond	2.3	
		F247	π stacking	~ 4.3	
**Honokiol**	**01**	F120	π stacking	~ 4.3	4.7
	**02**	F120	π stacking	~ 3.9	7.6
		S304	H-bond	2.5	
**Magnolol**	**01**	D301	ionic	1.9	6.0
		F120	π stacking	~ 4.0	
	**02**	D301	ionic	2.6	5.4
		S304	H-bond	2.3	
**Piperine**		F120	π stacking	~3.8	5.4
		F483	π stacking	~ 4.0	
**Protopine**		Q244	H-bond	2.4	5.9
		F120	π stacking	3.1	
		F120	π stacking	6.5	
		F483	π stacking	3.5	
**Scopoletin**		S304	H bond	2.4	5.3
		F120	π stacking	~ 3.8	
**Cannabidiol**	**01**	Q244	H-bond	2.3	4.7
		F120	π stacking	3.6	
	**02**	S304	H-bond	2.4	7.8
		F120	π stacking	~ 4.0	

TABLE S5 | Overview of the various types of interactions found in the best binding modes for CYP2D6*53.

Natural Product		4WNW V53		Distance	Heme
		AA	Type	(Å)	(Å)
Thymol		F120	π stacking	~3.8	4.7
s-auraptenol		D301	ionic	2.3	7.6
		S304	H-bond	2.4	
D-(-)-synephrine	01	D301	ionic	2.6	6.1
		F120	π-stacking	~ 4.7	
	02	E216	ionic	2.3	5.1
		Q244	H-bond	2.3	
		F247	π stacking	~ 4.3	
Honokiol	01	F120	π stacking	~ 4.3	4.7
	02	F120	π stacking	~ 3.9	7.6
		S304	H-bond	2.5	
Magnolol	01	D301	ionic	1.9	6.0
		F120	π stacking	~ 4.0	
	02	D301	ionic	2.6	5.4
		S304	H-bond	2.3	
Piperine		F120	π stacking	~3.8	5.4
		F483	π stacking	~ 4.0	
Protopine		Q244	H-bond	2.4	5.9
		F120	π stacking	3.1	
		F120	π stacking	6.5	
		F483	π stacking	3.5	
Scopoletin		S304	H bond	2.4	5.3
		F120	π stacking	~ 3.8	
Cannabidiol	01	Q244	H-bond	2.3	4.7
		F120	π stacking	3.6	
	02	S304	H-bond	2.4	7.8
		F120	π stacking	~ 4.0	

TABLE S6 | Overview QikProp descriptors with Schrödinger explanation

Descriptor	Explanation (Schrödinger – QikProp)
**log_{HERG}**	Predicted IC50 value for blockage of HERG K+ channels
**P_{Caco}**	Predicted apparent Caco-2 cell permeability in nm/sec. Caco2 cells are a model for the gut-blood barrier. QikProp predictions are for non-active transport.
**logBB**	Predicted brain/blood partition coefficient. Note: QikProp predictions are for orally delivered drugs so, for example, dopamine and serotonin are CNS negative because they are too polar to cross the blood-brain barrier
**P_{MDCK}**	Predicted apparent MDCK cell permeability in nm/sec. MDCK cells are considered to be a good mimic for the blood brain barrier (BBB). QikProp predictions are for non-active transport.
**HOA (%)**	Predicted human oral absorption on 0 to 100% scale. The prediction is based on a quantitative multiple linear regression model.
**CNS**	Predicted central nervous system activity on a –2 (inactive) to +2 (active) scale.
